# Digital Incoherent Compressive Holography Using a Geometric Phase Metalens

**DOI:** 10.3390/s21165624

**Published:** 2021-08-20

**Authors:** Jonghyun Lee, Youngrok Kim, Kihong Choi, Joonku Hahn, Sung-Wook Min, Hwi Kim

**Affiliations:** 1 Department of Electronics and Information Engineering, College of Science and Technology, Sejong-Campus, Korea University, 2511 Sejong-ro, Sejong 30019, Korea; iweks102@korea.ac.kr; 2 Department of Information Display, Kyung Hee University, 26 Kyungheedae-ro, Seoul 02447, Korea; faller825@khu.ac.kr (Y.K.); mins@khu.ac.kr (S.-W.M.); 3 Digital Holography Research Section, Electronics and Telecommunications Research Institute, 218 Gajeong-ro, Daejeon 34129, Korea; kihong08@etri.re.kr; 4 School of Electronic and Electrical Engineering, Kyungpook National University, 80 Daehak-ro, Daegu 41566, Korea; jhahn@knu.ac.kr

**Keywords:** diffractive optics, digital holography, computer-generated hologram, metasurface, compressive sensing, optical sectioning

## Abstract

We propose a compressive self-interference incoherent digital holography (SIDH) with a geometric phase metalens for section-wise holographic object reconstruction. We specify the details of the SIDH with a geometric phase metalens design that covers the visible wavelength band, analyze a spatial distortion problem in the SIDH and address a process of a compressive holographic section-wise reconstruction with analytic spatial calibration. The metalens allows us to realize a compressive SIDH system in the visible wavelength band using an image sensor with relatively low bandwidth. The operation of the proposed compressive SIDH is verified through numerical simulations.

## 1. Introduction

Digital holography is a key component of next-generation three-dimensional (3D) camera and imaging technology. Traditional digital holography employs the two-beamline interferometric system based on signal and reference arms from a highly coherent laser source [1,2,3,4,5,6]. However, coherent digital holography has limitations for many practical 3D applications because it cannot take 3D-scene holograms under daylight or incoherent illumination, such as that produced by light-emitting-diodes (LEDs).

To overcome these limitations, the basic schemes of recording and reconstructing the incoherent hologram were proposed by the earliest reports using the self-interference technique [7,8,9,10,11] to modulate incoherent object scenes into the holographic self-interfering patterns, and several related systems have been developed. The conventional interferometric structures such as with a rotational shearing [12,13,14], Mach–Zehnder [15], or Michelson interferometers [16,17] were employed in the system to spatially divide and modulate the incoming wavefront. Conoscopic holography was a method suggested to obtain the incoherent hologram without spatially dividing the incident object wavefront into two by utilizing the birefringent crystal slap [18]. With the development of opto-electronic image sensor devices, various types of SIDH systems have been used for a decade, in which the phase-only spatial light modulator (SLM) is adopted as a wavefront modulator [19,20,21]. In SIDH, the polarization state parallel to the active axis of SLM is modulated corresponding to the displayed quadratic phase pattern, and the orthogonal state just passes through the SLM, so that the Fresnel zone-like interference pattern can be obtained at the sensor plane. Besides using the phase-only SLM [22,23], the liquid crystal GRIN lens [24] or the birefringent crystal lens [25,26] were utilized as active-type wavefront modulating devices. The binary diffractive Fresnel zone lens [27], ring-shaped bifocal lens [28], bifocal metasurface lens [29], or the liquid crystal polymer-based polarization directed flat lens were implemented as passive-type wavefront modulating optics, giving rise to the simplicity of the system setup [30]. Most of the reported systems are described with the terms of self-interference incoherent digital holography (SIDH) or Fresnel incoherent correlation holography (FINCH). Since recording a hologram is performed using incoherent light, the design condition of incoherent holography is limited according to the condition that the optical path difference (OPD) must be smaller than the coherence length. However, the techniques SIDH and FINCH succeeded in becoming a main stream of incoherent holography by decreasing the restriction in the OPD systematically.

Choi et al. reported the incoherent digital holographic camera system with a liquid-crystal geometric phase (LCGP) lens using the in-line self-interference concept [31,32]. Furthermore, they succeeded in the compact implementation of an LCGP in-line system using a focal-point array with a micro-polarizer array embedded for real-time phase-shifting digital holography [33]. The system demonstrated the operation of capturing and generating incoherent holograms of real moving objects. However, the practical disadvantage of the LCGP lens is its low numerical aperture (NA) and chromatic aberration noise in the visible wavelength band. Furthermore, converging and diverging spherical wavefront inference in LCGP SIDH requires higher resolution CCD or CMOS devices, and this leads to limited spatial-frequency bandwidth or field-of-view (FOV).

The incoherent holographic system was developed to acquire holographic sectioning images [34,35]. In the FINCH scope scheme, the confocal imaging technique was combined to FINCH to obtain the optical sectioning capability [36]. Meanwhile, compressive holography [37,38] was proposed as a candidate for optical sectioning to overcome confocal optical sectioning. Through compressive holography, we can acquire the sectional images of the 3D target scene, excluding out-of-focus information on the other plane [37,38]. Recently, the compressive sensing (CS) algorithm was used to eliminate the twin and DC noise in the coaxial holographic system with a bifocal metalens [29]. The metalenses feature broadband 2π-phase modulation and can be made to have a high numerical aperture (NA) in the visible wavelength band. The geometric phase metalens [39,40,41,42] allows the design of ultra-compact SIDH optical systems by simultaneously interfering modulated and non-modulated waves exploiting its polarization selectivity.

This paper proposes incoherent compressive SIDH with a geometric phase metalens for section-wise holographic object reconstruction. We detail a compressive SIDH design by combining a geometric phase metalens and a compressive holography scheme. The proposed SIDH is shown to be capable of single-shot compressive holographic sectioning images. The correct depth information is crucial for the compressive holography-based optical sectioning [37,38]. As addressed in this paper, the incoherent holograms of 3D scenes captured by the SIDH system [30,31,32] have an issue regarding depth and x-y scale distortion. The phase of the object is not recorded as it is during the recording process. The spatial distortion correction of the difference between the reconstructed object from the incoherent hologram and the real object should be accounted for in the object reconstruction and depth information extraction of real 3D scenes. For this, the depth and lateral scale distortion along the optic axis of SIDH should be calibrated correctly.

## 2. SIDH with Geometric Phase Metalens and Spatial Distortion Compensation

### 2.1. SIDH System with a Geometric Phase Metalens

**Figure 1:**
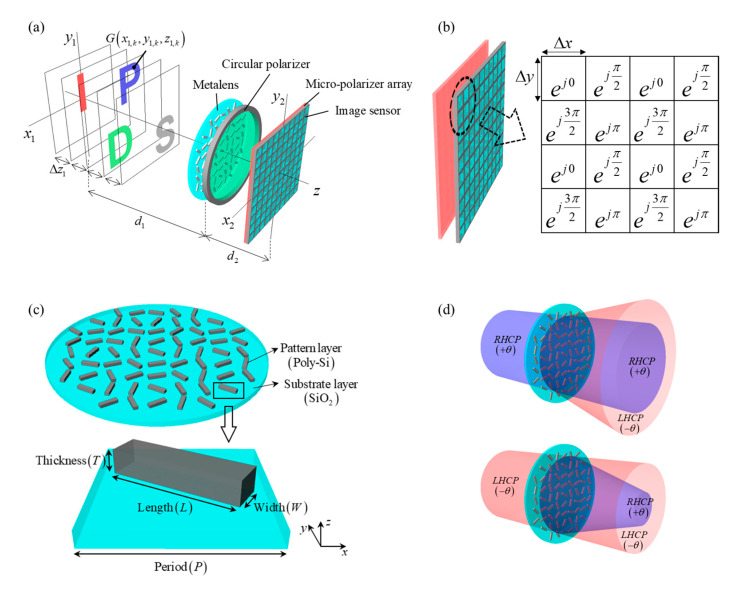
(**a**) The scheme of the self-interference incoherent digital holography (SIDH) system with metalens, (**b**) the image sensor with micro-polarizer array and the phase-shifted value δ of the corresponding matched subpixel, (**c**) the all-dielectric metalens and a unit pixel, and (**d**) the phase modulations of the metalens for the right-handed circular polarization (RHCP) and left-handed circular polarization (LHCP) incidence beams.

This section describes the SIDH system based on the polarization-sensitive geometric phase metalens and analyzes the distortion problem of that SIDH system. Figure 1a shows the scheme of the SIDH system with the metalens. The wave generated from a point on a target object propagates to the metalens, and the optical wave is split into two wave components by the metalens: the modulated cross-polarized converging wave and non-modulated co-polarized wave. As shown in Figure 1a, two wavefronts passing the circular polarizer generate the interference pattern in the form of a Fresnel zone plate (FZP) on the image sensor with a micro-polarizer array. The micro-polarizer plate is composed of a repeated 2 × 2 array with phase delay components of 0, π/4, π/2 and 3π/2, as shown in Figure 1b. A complex hologram is synthesized from the phase-shifted intensity images of the 3D scene [32,33]. The design of the unit pixel structure of the metasurface lens is depicted in Figure 1c.

The single nanorod pattern, which exerts a geometric phase effect by rotating an orientation angle, can be implemented in polycrystalline silicon on the silicon dioxide substrate, as illustrated in Figure 1c, where the period, length, width, and thickness of the rod are denoted by P, L, W, and T, respectively. Figure 1d describes the phase function of the metalens working selectively with respect to the polarization states of an incident wave. Through the geometric phase (GP) effect, the phase of the cross-polarization wave is modulated while the non-modulated co-polarization incident wave propagates normally in free space. The left-handed circular polarization (LHCP) incident wave is modulated with the convex lens phase function of the right-handed circular polarization (RHCP) wave component, while the RHCP incident wave is modulated by the concave lens phase function of the LHCP wave component.

If the incident wave with all polarization states is set without a polarizer in front of the metalens, the non-modulated wave of the RHCP incident wave and the modulated wave of the LHCP incident wave can be acquired from the interference pattern on the image sensor plane. The image sensor with the micro-polarizer array detects the interference pattern of the RHCP and LHCP components, and the complex hologram in the SIDH system is reconstructed by the phase-shifting holography technique. We next characterize the optical system in terms of the exact depth information of the 3D scene reconstructed from the complex hologram. For the analysis, let us set a depth-map object $G\left(x_{1},y_{1},z_{1}\right)$ and denote the k*th* layer image $G\left(x_{1,k},y_{1,k},z_{1,k}\right)$, as seen in Figure 1a. Under the incoherent illumination, the phase-shifted intensity image $H_{n}\left(x_{2},y_{2};\delta \right)$ on the image sensor is obtained as

(1) $$H_{n}\left(x_{2},y_{2};\delta \right)=\sum_{k}\iint {\left|G\left(x_{1,k},y_{1,k},z_{1,k}\right)\right|}^{2}I_{h}\left(x_{2},y_{2};x_{1,k},y_{1,k},z_{1,k},\delta \right)dx_{1,k}dy_{1,k}$$

where $I_{h}$ is the intensity pattern of the interference pattern for each point object, δ is the phase retardation of the polarized component [32,33]. $\left(x_{1,k},y_{1,k},z_{1,k}\right)$ is the coordinate of the kth point of the 3D object and $\left(x_{2},y_{2}\right)$ is the lateral coordinate on the image sensor.

As indicated in Figure 1d, the interference pattern $I_{h}$ of the point $\left(x_{1,k},y_{1,k},z_{1,k}\right)$ is taken by the interference of the converging wave component $Q_{1}$ and the non-modulated wave component $Q_{2}$, and it is given as

(2) $$I_{h}\left(x_{2},y_{2};x_{1,k},y_{1,k},z_{1,k},\delta \right)={\left|Q_{1}\left(x_{2},y_{2};x_{1,k},y_{1,k},z_{1,k},f\right)e^{-j\frac{\delta }{2}}+Q_{2}\left(x_{2},y_{2};x_{1,k},y_{1,k},z_{1,k}\right)e^{j\frac{\delta }{2}}\right|}^{2}$$

The propagator $Q_{1}$ of the modulated wave takes the form of the generalized Fresnel transform (GFrT) [43],

(3) $$\begin{array}{c} Q_{1}\left(x_{2},y_{2};x_{1,k},y_{1,k},z_{1,k},f\right)\\ =\frac{-j}{\left|\lambda \left\{\left(d_{1}-z_{1,k}\right)+d_{2}\right\}-\frac{\lambda \left(d_{1}-z_{1}\right)d_{2}}{f}\right|}\\ \times \exp\left[\frac{j\pi \left\{\left(1-\frac{\left(d_{1}-z_{1,k}\right)}{f}\right)\left(x_{2}^{2}+y_{2}^{2}\right)-2\left(x_{2}x_{1,k}+y_{2}y_{1,k}\right)+\left(1-\frac{d_{2}}{f}\right)\left(x_{1,k}^{2}+y_{1,k}^{2}\right)\right\}}{\lambda \left\{(d_{1}-z_{1,k})+d_{2}\right\}-\frac{\lambda (d_{1}-z_{1,k})d_{2}}{f}}\right] \end{array}$$

where f, $d_{1}$, $d_{2}$, and λ are the metalens’ focal length, the distance between the reference object plane and the metalens, the distance between the metalens and the image sensor, and the operating wavelength, respectively. The non-modulated wave component $Q_{2}$ takes the form of the Fresnel transform (FrT) [44],

(4) $$\begin{array}{l} Q_{2}\left(x_{2},y_{2};x_{1,k},y_{1,k},z_{1,k}\right)\\ =\frac{-j}{\left|\lambda \left\{\left(d_{1}-z_{1,k}\right)+d_{2}\right\}\right|}\\ \times \exp\left[\frac{j\pi \left\{\left(x_{2}^{2}+y_{2}^{2}\right)-2\left(x_{2}x_{1,k}+y_{2}y_{1,k}\right)+\left(x_{1,k}^{2}+y_{1,k}^{2}\right)\right\}}{\lambda \left\{(d_{1}-z_{1,k})+d_{2}\right\}}\right] \end{array}$$

Substituting Equations (3) and (4) into Equation (2), we obtain the interference pattern (see Appendix A):

(5) $$\begin{array}{c} I_{h}\left(x_{2},y_{2};x_{1,k},y_{1,k},z_{1,k}\right)\\ =\frac{1}{{\left|A\right|}^{2}}+\frac{1}{{\left|B\right|}^{2}}\\ -\frac{1}{\left|AB\right|}\times \exp\left[-\frac{j\pi \lambda {\left(d_{1}-z_{1,k}\right)}^{2}}{ABf}\left\{{\left(x_{2}+\frac{d_{2}}{d_{1}-z_{1,k}}x_{1,k}\right)}^{2}+{\left(y_{2}+\frac{d_{2}}{d_{1}-z_{1,k}}y_{1,k}\right)}^{2}\right\}\right]\times e^{-j\delta }\\ -\frac{1}{\left|AB\right|}\times \exp\left[\frac{j\pi \lambda {\left(d_{1}-z_{1,k}\right)}^{2}}{ABf}\left\{{\left(x_{2}+\frac{d_{2}}{d_{1}-z_{1,k}}x_{1,k}\right)}^{2}+{\left(y_{2}+\frac{d_{2}}{d_{1}-z_{1,k}}y_{1,k}\right)}^{2}\right\}\right]\times e^{j\delta }, \end{array}$$

where A and B are

(6) $$A=\lambda \left\{\left(d_{1}-z_{1,k}\right)+d_{2}\right\}-\frac{\lambda \left(d_{1}-z_{1,k}\right)d_{2}}{f}$$

and

(7) $$B=\lambda \left\{\left(d_{1}-z_{1,k}\right)+d_{2}\right\}$$

In the four-phase shifting holography scheme, the images of phase retardation (δ=0,π/2,π,3π/2) synthesize the final complex hologram (CH) pattern [33], represented by the form of the Fresnel transform of ${\left|G\left(x_{1,k},y_{1,k},z_{1,k}\right)\right|}^{2}$, (see Appendix B):

(8) $$\begin{array}{cc} \mathrm{CH} & =\left[H_{3}\left(x_{2},y_{2};\delta =\pi \right)-H_{1}\left(x_{2},y_{2};\delta =0\right)\right]-j\left[H_{4}\left(x_{2},y_{2};\delta =\frac{3\pi }{2}\right)-H_{2}\left(x_{2},y_{2};\delta =\frac{\pi }{2}\right)\right]\\ & =\sum_{k}\iint {\left|G\left(x_{1,k},y_{1,k},z_{1,k}\right)\right|}^{2}\frac{4\exp\left[\frac{j\pi }{\lambda {\bar{z}}_{1,k}}\left\{{\left(x_{2}-{\bar{x}}_{1,k}\right)}^{2}+{\left(y_{2}-{\bar{y}}_{1,k}\right)}^{2}\right\}\right]}{\left|\lambda^{2}{\left(d_{1}-z_{1,k}\right)}^{2}d_{2}^{2}\left(\frac{1}{d_{1}-z_{1,k}}+\frac{1}{d_{2}}-\frac{1}{f}\right)\left(\frac{1}{d_{1}-z_{1,k}}+\frac{1}{d_{2}}\right)\right|}dx_{1,k}dy_{1,k}, \end{array}$$

where ${\bar{x}}_{1,k}$, ${\bar{y}}_{1,k}$, and ${\bar{z}}_{1,k}$ are the new coordinates representing the distorted 3D scene. In this process, the nonlinear constant term and conjugate term of the hologram cancel out [33]. We have shown that the relationship between the complex hologram (CH) and the point cloud object G is based on the Fresnel transform of the distorted domain $\left({\bar{x}}_{1,k},{\bar{y}}_{1,k},d_{1}+d_{2}-{\bar{z}}_{1,k}\right)$. The distorted domain $\left({\bar{x}}_{1,k},{\bar{y}}_{1,k},d_{1}+d_{2}-{\bar{z}}_{1,k}\right)$ of the reconstructed object is identified as

(9) $$\begin{array}{l} \left({\bar{x}}_{1,k},{\bar{y}}_{1,k},d_{1}+d_{2}-{\bar{z}}_{1,k}\right)\\ =\left(-\frac{d_{2}^{}}{\left(d_{1}-z_{1,k}\right)}x_{1},-\frac{d_{2}^{}}{\left(d_{1}-z_{1,k}\right)}y_{1},d_{1}+d_{2}-d_{2}^{2}f\left(\frac{1}{d_{1}-z_{1,k}}+\frac{1}{d_{2}}-\frac{1}{f}\right)\left(\frac{1}{d_{1}-z_{1}}+\frac{1}{d_{2}}\right)\right) \end{array}$$

**Figure 2:**
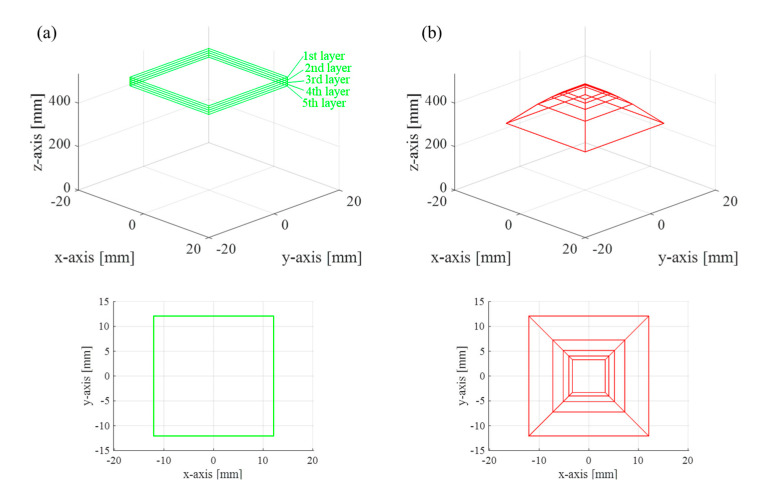
Spatial distortion relation of SIDH. (**a**) Target layered rectangle objects in the real space and (**b**) corresponding distorted objects reconstructed from the incoherent hologram recorded by the proposed SIDH system. The depth set along the *z*-axis of the real object is 520 mm, 510 mm, 500 mm, 490 mm, and 480 mm. The depth set of the reconstructed object is 307.5 mm, 396 mm, 427.8 mm, 443.9 mm, and 453.5 mm. The system parameters are set to $d_{1}=525\;\mathrm{mm}$, $d_{2}=15\;\mathrm{mm}$, and f=250 mm.

Equation (9) is the mapping relationship between the real and reconstructed spaces with distortion, where the multi-layered line objects in the real space are nonlinearly distorted in the reconstructed space. For instance, the real space object of five rectangular layers at 520 mm, 510 mm, 500 mm, 490 mm, and 480 mm on the *z*-axis is reconstructed to the distorted object of five layers at 307.5 mm, 396 mm, 427.8 mm, 443.9 mm, and 453.5 mm on the *z*-axis with lateral scale variation, as shown in Figure 2.

**Figure 3:**
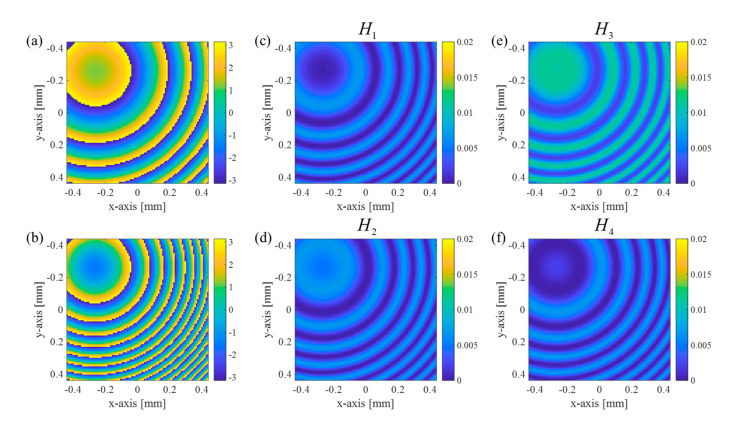
Phase profiles of complex field distributions on the image sensor: (**a**) the modulated wave (GFrT) and (**b**) the non-modulated wave (FrT). Phase shifted intensity profiles (**c**) $H_{1}$, (**d**) $H_{2}$, (**e**) $H_{3}$, and (**f**) $H_{4}$ with phase-shifted values 0,π/2,π,3π/2 simulated on the image sensor with micro-polarizer array. The point object is located at 30 mm depth and the system parameters are $d_{1}=70\;\mathrm{mm}$, $d_{2}=10\;\mathrm{mm}$, and f=100 mm.

Regarding the unit hologram pattern for an object point, six four-phase shifted hologram patterns, the modulated wavefront, the non-modulated wavefront, and the resulting $H_{1}$, $H_{2}$, $H_{3}$, and $H_{4}$ are presented in Figure 3.

From Equation (8), the hologram pattern of an object point $G\left(x_{1,k},y_{1,k},z_{1,k}\right)$ located at the kth layer is simplified to

(10) $${\mathrm{CH}}_{k}=4{\left|G\left(x_{1,k},y_{1,k},z_{1,k}\right)\right|}^{2}\frac{\exp\left[\frac{j\pi }{\lambda {\bar{z}}_{1,k}}\left\{{\left(x_{2}-{\bar{x}}_{1,k}\right)}^{2}+{\left(y_{2}-{\bar{y}}_{1,k}\right)}^{2}\right\}\right]}{\left|\lambda^{2}{\left(d_{1}-z_{1,k}\right)}^{2}d_{2}^{2}\left(\frac{1}{d_{1}-z_{1,k}}+\frac{1}{d_{2}}-\frac{1}{f}\right)\left(\frac{1}{d_{1}-z_{1,k}}+\frac{1}{d_{2}}\right)\right|}$$

Although the actual object point is $\left(x_{1,k},y_{1,k},z_{1,k}\right)$, ${\mathrm{CH}}_{k}$ is interpreted to a spherical wave coming from a different point $\left({\bar{x}}_{1,k},{\bar{y}}_{1,k},{\bar{z}}_{1,k}\right)$ in free space.

For a point cloud object, the coherent accumulation of the zone plate patterns of the point cloud object is recorded at the image sensor in the form of

(11) $$\mathrm{CH}=\sum_{k}{\mathrm{CH}}_{k}$$

**Figure 4:**
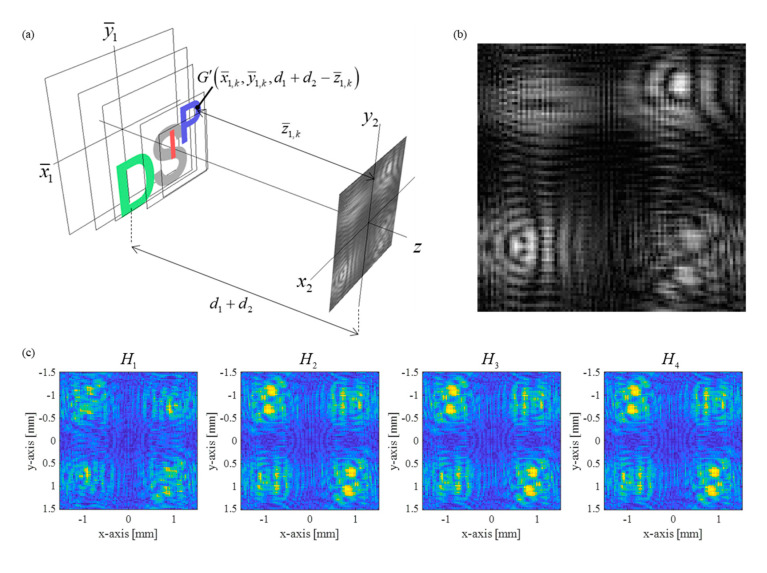
(**a**) The reconstruction scheme of the metalens SIDH, (**b**) the hologram pattern acquired by the metalens-based SIDH system ($d_{1}=90\;\mathrm{mm}$, $d_{2}=20\;\mathrm{mm}$, and f=120 mm), (**c**) four-phase shifted intensity profiles of all object points recorded on the image sensor. The image sensor is assumed to have 101 by 101 resolution, 30 μm pixel pitch, and the operating wavelength of 550 nm.

Accordingly, the complex hologram of the multi-depth letter image set in Figure 1a is reconstructed to the distorted objects, as illustrated in Figure 4a,b.

While the depth $z_{1,k}$ of letters ‘I’, ‘P’, ‘D’, and ‘S’ are 10 mm, 20 mm, 30 mm, and 40 mm measured from the zero-depth plane $\left(z_{1}=0\right)$, the estimated in-focus depths ${\bar{z}}_{1,k}$ corresponding to each letter are measured as 162.5 mm, 172.7 mm, 186.7 mm, and 207.2 mm. Note that the issue is to investigate whether the 3D objects can be reconstructed in the real 3D space correctly. We believe that optical sectioning is necessary to deal with this problem and, in the next section, test the compressive holography operation in the proposed metalens-based SIDH scheme. For all object points, four-phase shifted intensity profiles $H_{1}$, $H_{2}$, $H_{3}$, and $H_{4}$ according to Equation (1) are presented in Figure 4c. We assume that the image sensor has a 101 by 101 resolution, 30 μm pixel pitch, and the operating wavelength of 550 nm. The shifted phase values 0, π/2, π, and 3π/2 are the same in Figure 3. Additionally, the image sensor is assumed to be the ideal situation where the subpixels are matched spatially.

**Figure 5:**
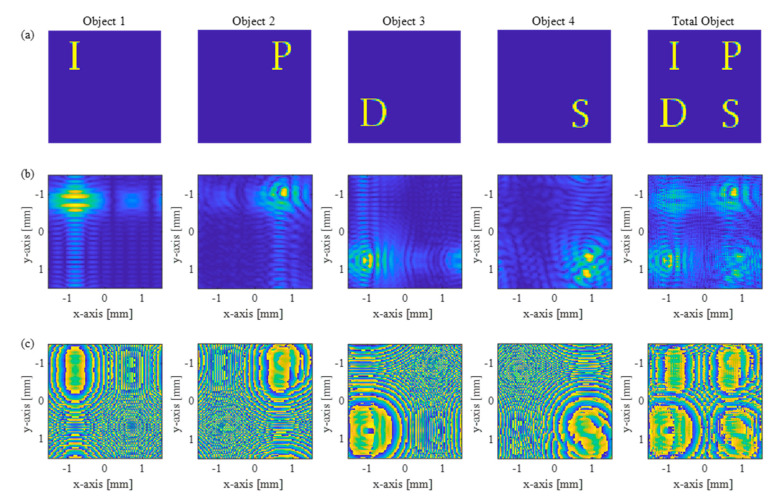
The complex hologram by the proposed scheme. (**a**) Four letter target objects of ‘I’, ‘P’, ‘D’, and ‘S’ where their depths from the image sensor (Hologram plane) are set to (100 mm, 90 mm, 80 mm, and 70 mm). (**b**) Amplitude profiles and (**c**) phase profiles of each incoherent complex hologram synthesized by the SIDH system.

Figure 5a–c show the target objects, the amplitude profiles, and phase profiles of the complex holograms of the SIDH system based on a metalens. We set the images of four target objects in the form of letters ‘I’, ‘P’, ‘D’, and ‘S’ in Figure 5a, which are 100 mm, 90 mm, 80 mm, and 70 mm, respectively, from the image sensor. Then, the results of the complex holograms are computed according to Equation (8) for each depth distance, and their amplitude profiles and phase profiles on the image sensor are shown in Figure 5b,c. The data sets presented from the first column to the fourth column are the cases for each object, and the data presented in the last column are the total holograms calculated for all objects.

**Figure 6:**
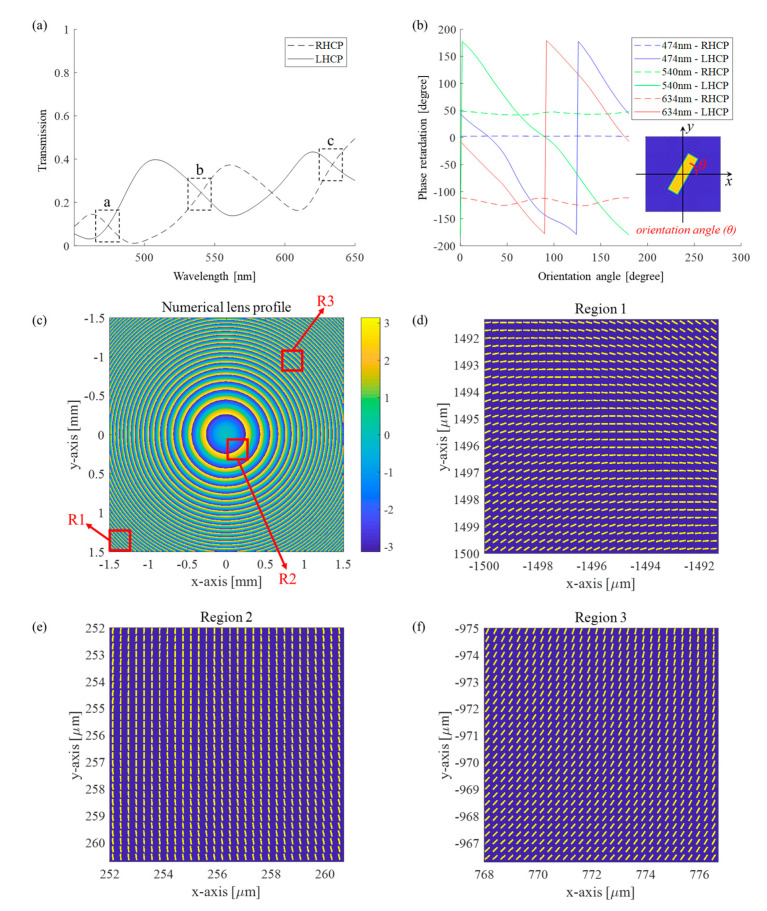
Light modulation characteristics of the metalens. (**a**) The transmission efficiency and (**b**) the phase modulation characteristics of the RHCP cross-polarization state and LHCP co-polarization of (a) the metalens in the visible band. Equal transmission efficiency of the RHCP and LHCP states are obtained at point a (474 nm), b (540 nm), and c (634 nm). (**c**) Numerical phase profile of the metalens lens with a focal length 120 mm, and the metasurface pixel distributions in each region which are (**d**) region 1 (R1), (**e**) region 2 (R2), and (**f**) region 3 (R3).

It is noteworthy that the intensity of the modulated component and the non-modulated component should be similar in order to acquire a balanced interference pattern. To achieve this, an optimally designed metalens is essential. The modulation characteristics of the nanorod structure are analyzed using the Fourier modal method (FMM) [45]. In the analysis, the polarization state of the incident wave is assumed to be RHCP. The optimal design parameters of the metasurface structure were determined through the parametric study and the resulting structural parameters: the period P, length L, width W, and thickness T of the poly-Si nanorod are 300 nm, 250 nm, 60 nm, and 150 nm, respectively. The operational transmission characteristics of the designed nanorod are shown in Figure 6. The metasurface structure has broadband characteristics in the visible wavelength range from 450 nm to 650 nm. Figure 6a presents the transmission efficiency of the RHCP and LHCP components for wavelengths in the visible band.

The wavelengths indicated by the boxes a, b, and c correspond to the central wavelengths of red (634 nm), green (540 nm), blue (474 nm), confirming the almost equal diffraction efficiencies of RHCP and LHCP. In Figure 6b, the geometric phase modulation characteristics of RHCP and LHCP for the three central wavelengths are plotted with respect to the orientation angle of the nanorod. The dotted lines indicate the constant flat phase modulation of the non-modulated co-polarized wave component, while 2π full-range phase modulation of the cross-polarization wave component is clarified by the solid lines for every wavelength. In Figure 6c, the phase profile of the numerical convex lens is presented. The metasurface for each local region is designed by the phase that can act as a lens, which can be designed based on the GP effect of the metasurface, as shown in Figure 6d–f. The orientation angle of rod for each subpixel can be designed according to the results of phase modulation presented in Figure 6b. Considering this broadband performance of the metalens, we can expect that the metalens can be more effective at recording incoherent visible band holograms than the LC-based GP lens. Furthermore, if a low-loss dielectric material such as titanium dioxide or silicon nitride was used for the metalens, the transmission efficiency of the metalens would be increased.

### 2.2. Compressive Holography

We employ compressive holography [37,38] as an essential step of correct 3D scene measurement. Compressive holography in the proposed metalens-based SIDH scheme highly depends on accurate depth estimation. We compare the compressive holography sectioning of the cases with and without the depth estimation correction.

To acquire the section-wise field distribution of each object wave, we apply compressive sensing (CS) based on the two-step iterative shrinkage/thresholding (TwIST) algorithm [46] to the hologram recorded by the SIDH system. In the previous section, the complex hologram (CH) was expressed through Equation (8) as the Fresnel transform of the point object ${\left|G\left(x_{1,k},y_{1,k},z_{1,k}\right)\right|}^{2}$, and further simplified to

(12) $$\mathrm{CH}=\sum_{k}FrT_{k}\left\{{\left|G\left(x_{1,k},y_{1,k},z_{1,k}\right)\right|}^{2}\right\}$$

where $FrT_{k}\left\{\cdot \right\}$ denotes the $z_{1,k}$ -depth-specific Fresnel transform defined on the distorted domain. Let a two-dimensional (2D) complex hologram (CH) of $N_{x}\times N_{y}$ resolution and Δx×Δy pixel pitch convert to the one-dimensional (1D) vector g as

(13) $$g_{\left(n-1\right)\times N_{x}+m}=\mathrm{CH}\left(m\Delta x,n\Delta y\right)$$

Let the 3D object data of $N_{x}\times N_{y}\times N_{z}$ resolution and Δx×Δy×Δz pixel pitch convert to the 1D vector f, represented as

(14) $$f_{\left(l-1\right)\times N_{x}\times N_{y}+\left(n-1\right)\times N_{x}+m}=G\left(m\Delta x,n\Delta y,l\Delta z\right)$$

In the compressive sensing (CS) process, Equation (12) can be rewritten as

(15) g=B⋅I⋅Ff=Hf

where B and I denote the inverse Fresnel transform matrix defined on the distorted domain and $I=\left[I_{h,1}I_{h,2}\cdot \cdot \cdot I_{h,N_{z}}\right]$ with the intensity profile $I_{h,l}=I_{h}\left(x_{2},y_{2};x_{1,l},y_{1,l},z_{1,l}\right)$ of Equation (5), and F is the block diagonal matrix of depth-specific $FrT_{k}$ $F=bldiag\left(FrT_{1},\cdot \cdot \cdot ,FrT_{k},\cdot \cdot \cdot ,FrT_{K}\right)$. H is set as the measurement matrix of the compressive sensing operator of the SIDH system [37,38]. The forward transformation model of Equation (15) is inverted by decompressive inference using the TwIST algorithm such that

(16) $$\hat{f}=\underset{f}{\arg\min}{\left\|f\right\|}_{TV}$$

where $\hat{f}$ is the field distribution estimated by the TwIST algorithm, and ‖⋅‖ denotes $L_{0}$ -norm, and ${\left\|\cdot \right\|}_{TV}$ denotes the total variation [47].

We can estimate the accurate depth of the object layer using backpropagation reconstruction of the complex hologram and sequentially extract the section-wise field distribution by exploiting the TwIST algorithm and the estimated depth information.

## 3. Results and Discussion

The hologram reconstruction at a particular depth is classified into two branches: backpropagation-based field reconstruction and compressive holography-based optical sectioning, both at the same depth. Backpropagation-based reconstruction and compressive holography-based optical sectioning are tested against each other for the cases using the original depth set (100 mm, 90 mm, 80 mm, and 70 mm) (Figure 1a) and the compensated depth set (162.5 mm, 172.7 mm, 186.7 mm, and 207.2 mm) (Figure 4a). The former is the depth set without consideration of the system distortion analysis, that is the original depth set of the target image ‘I’,’P’,’D’, and ‘S’. The latter is the depth set modified by the distortion analysis.

**Figure 7:**
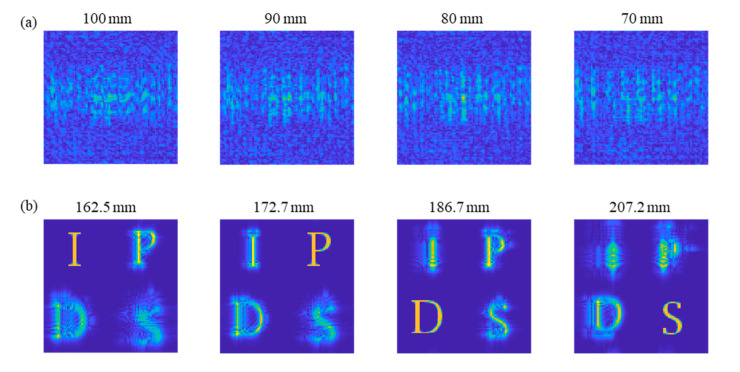
Back-propagated field distribution of the hologram (**a**) for the depth information (100 mm, 90 mm, 80 mm, and 70 mm) without distortion compensation, and (**b**) with distortion compensation (162.5 mm, 172.7 mm, 186.7 mm, and 207.2 mm) (Each column from the left presents the result of object ‘I’, ‘P’, ‘D’, and ‘S’).

Figure 7a,b present the simulation results of reconstructing the field distribution at each depth of the two depth sets, respectively. For the original depth set, incorrect reconstructed field distribution is acquired if real depth distances are applied. It is shown that clear target images are not perceived due to out-of-focus diffraction effect images.

**Figure 8:**
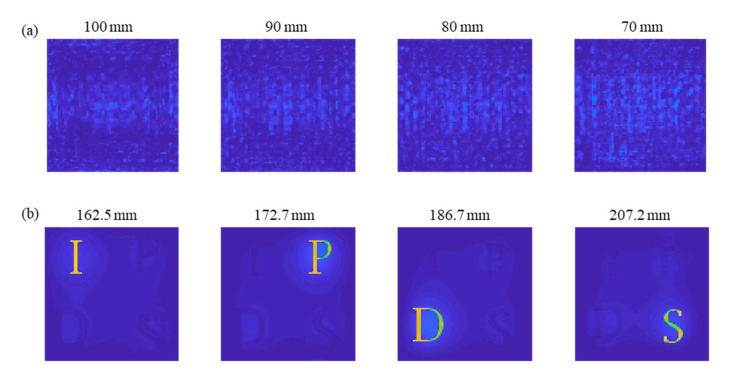
Compressive holographic sectioning of the hologram (**a**) for the depth information (100 mm, 90 mm, 80 mm, and 70 mm). without distortion compensation, and (**b**) with distortion compensation (162.5 mm, 172.7 mm, 186.7 mm, and 207.2 mm). (Each column from the left presents the result of object ‘I’, ‘P’, ‘D’, and ‘S’).

In contrast, by applying this estimated depth information, we can acquire the correct back-propagated field in Figure 7b. The target object is clearly observed at the second depth set. The parametric investigation on the reconstructed field distribution enables the practical estimation of correct depth information. However, the correct 3D object reconstruction is further processed because of the lateral scale change indicated by Equation (9). Therefore, for complete 3D reconstruction, we need to correct the lateral scale distortion of the object, as well as the depth distortion. Achieving this necessitates optical sectioning. Figure 8a,b compare the numerical optical sectioning results of the same complex hologram using TwIST compressive sensing with the original depth set and the corrected depth set, respectively.

The use of the original depth information of the target object acquires an incorrect section-wise reconstructed field distribution, as shown in Figure 8a. However, by applying the accurate depth distances computed for each layer by Equation (9), section-wise images can be observed clearly without other out-of-focus images in Figure 8b. Because the obtained section image is scaled in the distorted domain $\left(\bar{x},\bar{y}\right)$, the sectioned field distribution is post-processed through the lateral scaling of Equation (9) to correctly reform the layered 3D object in the real-world domain (x,y). Through Figure 7 and Figure 8, we show the results of testing the mathematical modeling of the metalens SIDH system and the influence of the depth parameters on the holographic reconstruction.

## 4. Conclusions

In conclusion, we proposed the design and developed the mathematical model of self-interference incoherent digital holography using a geometric phase metalens. The compressive holographic reconstruction scheme based on the model was tested and the features of the distortion mapping between the real object space and the distorted reconstruction space related to the holographic reconstruction were clarified. To increase the efficiency of compressive sensing (CS) sectioning, the polarization-sensitive geometric phase lens is required to be of low F-number and broadband-workable. A critical issue in the design of nanorod-based GP metalenses is ensuring the co-polarized and cross-polarized scattering powers are evenly distributed in the wavelengths in the red, green, and blue range. We have shown that the GP metalens supports the required broadband operation in the visible band. It is thought that the metalens is more competitive in terms of high numerical aperture (NA) and broad bandwidth compared to the conventional LC GP lens. Since SIDH inherently involves spatial distortion, we presented the compressive holographic sectioning process of a multi-layered target object with distortion compensation analysis. We anticipate that incoherent holographic camera technology will become a prevailing 3D imaging technology for a variety of applications in the near future, and that the proposed design and analysis will contribute both theoretical perspectives and practical techniques to the field of incoherent 3D imaging.

## Data Availability

Data sharing not applicable.

## Appendix A. The Intensity of Interference Pattern for a Single Point Source

The intensity of the interference pattern of a single point source $I_{h}$ is represented by

(A1) $$\begin{array}{cc} I_{h}\left(x_{2},y_{2};x_{1,k},y_{1,k},z_{1,k}\right) & ={\left|Q_{1}\left(x_{2},y_{2};x_{1,k},y_{1,k},z_{1,k};f\right)e^{-j\frac{\delta }{2}}+Q_{2}\left(x_{2},y_{2};x_{1,k},y_{1,k},z_{1,k}\right)e^{j\frac{\delta }{2}}\right|}^{2}\\ & =Q_{1}\left(x_{2},y_{2};x_{1,k},y_{1,k},z_{1,k};f\right)\times Q_{1}{}^{*}\left(x_{2},y_{2};x_{1,k},y_{1,k},z_{1,k};f\right)\\ & +Q_{2}\left(x_{2},y_{2};x_{1,k},y_{1,k},z_{1,k}\right)\times Q_{2}{}^{*}\left(x_{2},y_{2};x_{1,k},y_{1,k},z_{1,k}\right)\\ & +Q_{1}\left(x_{2},y_{2};x_{1,k},y_{1,k},z_{1,k};f\right)\times Q_{2}{}^{*}\left(x_{2},y_{2};x_{1,k},y_{1,k},z_{1,k}\right)\times e^{-j\delta }\\ & +Q_{2}\left(x_{2},y_{2};x_{1,k},y_{1,k},z_{1,k}\right)\times Q_{1}{}^{*}\left(x_{2},y_{2};x_{1,k},y_{1,k},z_{1,k};f\right)\times e^{j\delta } \end{array}$$

Four terms can be represented as

(A2) $$Q_{1}\left(x_{2},y_{2};x_{1,k},y_{1,k},z_{1,k};f\right)\times Q_{1}{}^{*}\left(x_{2},y_{2};x_{1,k},y_{1,k},z_{1,k};f\right)=\frac{1}{{\left|A\right|}^{2}}$$

(A3) $$Q_{2}\left(x_{2},y_{2};x_{1,k},y_{1,k},z_{1,k}\right)\times Q_{2}{}^{*}\left(x_{2},y_{2};x_{1,k},y_{1,k},z_{1,k}\right)=\frac{1}{{\left|B\right|}^{2}}$$

(A4) $$\begin{array}{c} Q_{1}\left(x_{2},y_{2};x_{1,k},y_{1,k},z_{1,k};f\right)\times Q_{2}{}^{*}\left(x_{2},y_{2};x_{1,k},y_{1,k},z_{1,k}\right)\times e^{-j\delta }\\ =\frac{-1}{\left|AB\right|}\exp\left[-\frac{j\pi \lambda }{AB}\left(\frac{{\left(d_{1}-z_{1,k}\right)}^{2}}{f}\right)\left\{{\left(x_{2}+\frac{d_{2}}{d_{1}-z_{1,k}}x_{1,k}\right)}^{2}+{\left(y_{2}+\frac{d_{2}}{d_{1}-z_{1,k}}y_{1,k}\right)}^{2}\right\}\right]\times e^{-j\delta } \end{array}$$

(A5) $$\begin{array}{c} Q_{2}\left(x_{2},y_{2};x_{1,k},y_{1,k},z_{1,k}\right)\times Q_{1}{}^{*}\left(x_{2},y_{2};x_{1,k},y_{1,k},z_{1,k};f\right)\times e^{j\delta }\\ =\frac{-1}{\left|AB\right|}\exp\left[\frac{j\pi \lambda }{AB}\left(\frac{{\left(d_{1}-z_{1,k}\right)}^{2}}{f}\right)\left\{{\left(x_{2}+\frac{d_{2}}{d_{1}-z_{1,k}}x_{1,k}\right)}^{2}+{\left(y_{2}+\frac{d_{2}}{d_{1}-z_{1,k}}y_{1,k}\right)}^{2}\right\}\right]\times e^{j\delta } \end{array}$$

Substituting Equations (A2)–(A5) into Equation (A1), we can obtain

(A6) $$\begin{array}{c} I_{h}\left(x_{2},y_{2};x_{1,k},y_{1,k},z_{1,k}\right)\\ =\frac{1}{{\left|A\right|}^{2}}+\frac{1}{{\left|B\right|}^{2}}\\ -\frac{1}{\left|AB\right|}\times \exp\left[-\frac{j\pi \lambda {\left(d_{1}-z_{1,k}\right)}^{2}}{ABf}\left\{{\left(x_{2}+\frac{d_{2}}{d_{1}-z_{1,k}}x_{1,k}\right)}^{2}+{\left(y_{2}+\frac{d_{2}}{d_{1}-z_{1,k}}y_{1,k}\right)}^{2}\right\}\right]\times e^{-j\delta }\\ -\frac{1}{\left|AB\right|}\times \exp\left[\frac{j\pi \lambda {\left(d_{1}-z_{1,k}\right)}^{2}}{ABf}\left\{{\left(x_{2}+\frac{d_{2}}{d_{1}-z_{1,k}}x_{1,k}\right)}^{2}+{\left(y_{2}+\frac{d_{2}}{d_{1}-z_{1,k}}y_{1,k}\right)}^{2}\right\}\right]\times e^{j\delta } \end{array}$$

where A and B can be represented as

(A7a) $$A=\lambda \left\{\left(d_{1}-z_{1,k}\right)+d_{2}\right\}-\frac{\lambda \left(d_{1}-z_{1,k}\right)d_{2}}{f}$$

(A7b) $$B=\lambda \left\{\left(d_{1}-z_{1,k}\right)+d_{2}\right\}$$

## Appendix B. The Total Intensity Profile$H_{n}$ by Phase Shifting δ

For the point object $G\left(x_{1,k},y_{1,k},z_{1,k}\right)$ on the k*th* target object plane, the phase-shifted image $H_{n}\left(x_{2},y_{2};\delta \right)$ on the image sensor is represented as

(A8) $$\begin{array}{c} H_{n}\left(x_{2},y_{2};\delta \right)\\ =\sum_{k}\iint {\left|G\left(x_{1,k},y_{1,k},z_{1,k}\right)\right|}^{2}I_{h}\left(x_{2},y_{2};x_{1,k},y_{1,k},z_{1,k},\delta \right)dx_{1,k}dy_{1,k}\\ =\sum_{k}\iint {\left|G\left(x_{1,k},y_{1,k},z_{1,k}\right)\right|}^{2}\\ \times [\frac{1}{{\left|A\right|}^{2}}+\frac{1}{{\left|B\right|}^{2}}\\ -\frac{1}{\left|AB\right|}\times \exp\left[-\frac{j\pi \lambda {\left(d_{1}-z_{1,k}\right)}^{2}}{ABf}\left\{{\left(x_{2}+\frac{d_{2}}{d_{1}-z_{1,k}}x_{1,k}\right)}^{2}+{\left(y_{2}+\frac{d_{2}}{d_{1}-z_{1,k}}y_{1,k}\right)}^{2}\right\}\right]\times e^{-j\delta }\\ -\frac{1}{\left|AB\right|}\times \exp\left[\frac{j\pi \lambda {\left(d_{1}-z_{1,k}\right)}^{2}}{ABf}\left\{{\left(x_{2}+\frac{d_{2}}{d_{1}-z_{1,k}}x_{1,k}\right)}^{2}+{\left(y_{2}+\frac{d_{2}}{d_{1}-z_{1,k}}y_{1,k}\right)}^{2}\right\}\right]\times e^{j\delta }]dx_{1,k}dy_{1,k} \end{array}$$

The phase shifted values for each polarization state of the image sensor with micro-polarizer are 0, π/2, π, and 3π/2. Hence, four total intensity profiles $H_{1}$, $H_{2}$, $H_{3}$, and $H_{4}$ are represented by $H_{1}\left(x_{2},y_{2};\delta =0\right)$, $H_{2}\left(x_{2},y_{2};\delta =\frac{\pi }{2}\right)$, $H_{3}\left(x_{2},y_{2};\delta =\pi \right)$, and $H_{4}\left(x_{2},y_{2};\delta =\frac{3\pi }{2}\right)$.

The synthesized complex hologram (CH) with the four total intensity profiles $H_{1}$, $H_{2}$, $H_{3}$, and $H_{4}$ is defined as

(A9) $$\begin{array}{cc} CH & =\left[H_{3}\left(x_{2},y_{2};\delta =\pi \right)-H_{1}\left(x_{2},y_{2};\delta =0\right)\right]\\ & -j\left[H_{4}\left(x_{2},y_{2};\delta =\frac{3\pi }{2}\right)-H_{2}\left(x_{2},y_{2};\delta =\frac{\pi }{2}\right)\right] \end{array}$$

The real part of CH is represented as

(A10) $$\begin{array}{c} H_{3}\left(x_{2},y_{2};\delta =\pi \right)-H_{1}\left(x_{2},y_{2};\delta =0\right)\\ ={∭\left|G\left(x_{1},y_{1},z_{1}\right)\right|}^{2}\\ \times [\frac{2}{\left|AB\right|}\times \exp\left[-\frac{j\pi \lambda {\left(d_{1}-z_{1,k}\right)}^{2}}{ABf}\left\{{\left(x_{2}+\frac{d_{2}}{d_{1}-z_{1,k}}x_{1,k}\right)}^{2}+{\left(y_{2}+\frac{d_{2}}{d_{1}-z_{1,k}}y_{1,k}\right)}^{2}\right\}\right]\\ +\frac{2}{\left|AB\right|}\times \exp\left[\frac{j\pi \lambda {\left(d_{1}-z_{1,k}\right)}^{2}}{ABf}\left\{{\left(x_{2}+\frac{d_{2}}{d_{1}-z_{1,k}}x_{1,k}\right)}^{2}+{\left(y_{2}+\frac{d_{2}}{d_{1}-z_{1,k}}y_{1,k}\right)}^{2}\right\}\right]]dx_{1,k}dy_{1,k} \end{array}$$

The imaginary part of CH is represented as

(A11) $$\begin{array}{c} H_{4}\left(x_{2},y_{2};\delta =\frac{3\pi }{2}\right)-H_{2}\left(x_{2},y_{2};\delta =\frac{\pi }{2}\right)\\ ={∭\left|G\left(x_{1},y_{1},z_{1}\right)\right|}^{2}\\ \times [\frac{-2j}{\left|AB\right|}\times \exp\left[-\frac{j\pi \lambda {\left(d_{1}-z_{1,k}\right)}^{2}}{ABf}\left\{{\left(x_{2}+\frac{d_{2}}{d_{1}-z_{1,k}}x_{1,k}\right)}^{2}+{\left(y_{2}+\frac{d_{2}}{d_{1}-z_{1,k}}y_{1,k}\right)}^{2}\right\}\right]\\ +\frac{2j}{\left|AB\right|}\times \exp\left[\frac{j\pi \lambda {\left(d_{1}-z_{1,k}\right)}^{2}}{ABf}\left\{{\left(x_{2}+\frac{d_{2}}{d_{1}-z_{1,k}}x_{1,k}\right)}^{2}+{\left(y_{2}+\frac{d_{2}}{d_{1}-z_{1,k}}y_{1,k}\right)}^{2}\right\}\right]]dx_{1,k}dy_{1,k} \end{array}$$

Substituting Equations (A10) and (A11) into Equation (A9), we can obtain

(A12) $$\begin{array}{cc} \mathrm{CH} & ={∭\left|G\left(x_{1,k},y_{1,k},z_{1,k}\right)\right|}^{2}\\ & \times \frac{4}{\left|AB\right|}\exp\left[\frac{j\pi \lambda {\left(d_{1}-z_{1,k}\right)}^{2}}{ABf}\left\{{\left(x_{2}+\frac{d_{2}}{d_{1}-z_{1,k}}x_{1,k}\right)}^{2}+{\left(y_{2}+\frac{d_{2}}{d_{1}-z_{1,k}}y_{1,k}\right)}^{2}\right\}\right]dx_{1,k}dy_{1,k}\\ & ={∭\left|G\left(x_{1,k},y_{1,k},z_{1,k}\right)\right|}^{2}\\ & \times \frac{4\exp\left[\frac{j\pi }{\lambda {\bar{z}}_{1,k}}\left\{{\left(x_{2}-{\bar{x}}_{1,k}\right)}^{2}+{\left(y_{2}-{\bar{y}}_{1,k}\right)}^{2}\right\}\right]}{\left|\lambda^{2}{\left(d_{1}-z_{1,k}\right)}^{2}d_{2}^{2}\left(\frac{1}{d_{1}-z_{1,k}}+\frac{1}{d_{2}}-\frac{1}{f}\right)\left(\frac{1}{d_{1}-z_{1,k}}+\frac{1}{d_{2}}\right)\right|}dx_{1,k}dy_{1,k} \end{array}$$

For Equation (A12), the distortion relation is obtained as

(A13) $$\begin{array}{l} \left({\bar{x}}_{1,k},{\bar{y}}_{1,k},d_{1}+d_{2}-{\bar{z}}_{1,k}\right)\\ =\left(-\frac{d_{2}}{d_{1}-z_{1,k}}x_{1,k},-\frac{d_{2}}{d_{1}-z_{1,k}}y_{1,k},d_{1}+d_{2}-fd_{2}^{2}\left(\frac{1}{d_{1}-z_{1,k}}+\frac{1}{d_{2}}-\frac{1}{f}\right)\left(\frac{1}{d_{1}-z_{1,k}}+\frac{1}{d_{2}}\right)\right) \end{array}$$
